# Challenges of Establishing a National Rare Donor Program in Iran

**Published:** 2018-07-01

**Authors:** Behrooz Ghezelbash, Mostafa Moghaddam, Sima Aghazadeh

**Affiliations:** 1 Laboratory Hematology & Blood Bank, Blood Transfusion Research Center, High Institute for Research and Education in Transfusion Medicine, Tehran, Iran; 2 Head of Department of Immunohematology, Blood Transfusion Research Center, High Institute for Research and Education in Transfusion Medicine, Tehran, Iran; 3 Ardabil University of Medical Sciences, Ardabil, Iran

**Keywords:** Rare blood program, Rare blood type, Rare donor

## Abstract

**Background: **Over the past decades, interest in establishing a National Rare Donor Program has increased significantly worldwide. The experience of developing countries, however, is still limited. Rare blood is defined as a blood group found in a 1000- 5000 population and donor has an absence of a high-prevalence antigen, or the absence of multiple common antigens. Iranian national rare donor program was established in 2009. This paper reports the experiences and challenges of establishing a national rare donor program in Iran.

**Materials and Methods: **This program provides services to all medical centers that need rare units. The main role of rare donor program is to maintain information of rare donors that are identified at the immunohematology reference laboratory located in Tehran. Good manufacturing practices and standard operating procedures are utilized to all activity. The IRL secures frozen blood to make them available when rare blood is required.

**Results: **As many as 1000 different types of rare donors have been identified in Iran, including several individuals whose blood group had developed clinically significant allo-antibodies. In addition to routine donors' personally identifiable information such as addresses and telephone numbers, we also access to the contact information of their close relatives or friends for emergency situation. Contact data are kept up to date at least twice annually. IRL staff are ready to provide services to patients with rare blood types, 24 hours per day, 7 days per week. To date, more than 80 donors with very rare blood group are listed on the IRL rare donor database in 31 centers. Current practice at IRL is to screen the first and second-degree relatives of any patient found to have a rare blood type for a matching blood donor. Iranian blood services need to establish special departments to provide rare blood RBCs and technical assistance for a quicker and more efficient responses to patients and request of their medical staff for blood transfusion. To achieve this aim, there were several challenges, including situation analysis and justification of the program, allocation of financial support by top managers, engineering and technical maintenance, facility and environmental services, employee awareness and communication between blood centers, technologist training in advanced immunohematology.

**Conclusion: **The results of this survey are encouraging and indicate that the information and database for rare donors will provide services to patients with very difficult and complex serology test results requiring rare blood transfusion. The experience of IRL may be helpful for other transfusion centers in developing countries.

## Introduction

 For many years, one of the challenges in transfusion medicine was to ensure the availability of compatible units of rare blood for patients with either single or multiple blood group antibodies present^1^. These rare blood types may represent a significant impediment to both urgent and planned transfusion of red blood cells (RBCs) and also carry out important administrative and clinical challenges for the practitioner. Clinical guidelines address little about the management of patients with unique blood group. Patients who require blood from donors known as “rare blood donors” present unique challenges both at the preoperative health care delivery system at large and for the individual clinician who desires to maintain safe, efficient, and cost-effective care. It is most challenging to supply blood for a patient with an antibody to an antigen of high prevalence, with an occurrence rate of less than one in 1000^2^ Often, there are other antibodies against common blood group antigens present with antibodies to high prevalence antigens, which provide a higher challenge^3^. Blood banks should answer the following questions:

How frequently one is expected to encounter these patients? How frequently does a non-ABO hemolytic transfusion reaction occur if an alloimmunized patient is transfused on an emergent basis? What effect will be the advent of red cell molecular genotyping played in providing blood for patients with rare blood types? Are regional and national rare donor programs often able to cope with the majority of requests for rare blood RBC units? Unfortunately, few medical centers are aware of the existence of the program that organizes information about rare blood donors and seeks to assist providing rare blood. This article introduces activity of the IRL and challenges of establishing a national rare donor program in Iran with task to answer the above questions.

**History of pioneering rare blood activities**

The need for a rare donor registry was noted at an early stage of blood transfusion development. The UK blood group reference laboratory was established in 1946 for the national blood transfusion service^3^. By 1959, the AABB had improved the rare donor file, and the American Red Cross created the rare donor registry. Before merging their programs in 1998, the ARC and AABB maintained separate rare donor databases and separate programs. About the same time, a similar panel was established in France to meet local needs^4^. 

The International Panel of Rare Blood Donors or International Donor Panel (IDP) was formed in 1965 under the directive of the ISBT and the day to day running of the IDP has been carried out by the red cell reference department of the International Blood Group Reference Laboratory (IBGRL) since that time^5^. The ISBT rare blood donor working party (ISBT WP) was appointed in 1985 by the ISBT to provide oversight of any matters related to the supply of rare blood donations on an international basis^1^^,^^4^^,^^5^. 

**Chain of activities at Iranian National Rare Donor Program**

Iranian blood transfusion organization (IBTO) was established in 1974. Thirty-five years later, Iranian national rare donor program was established in 2009. The central Iranian immunohaematology reference laboratory (IRL) has the responsibility of identification of donors with rare types and maintaining the database of rare blood groups in Tehran. Moreover, it is working to provide services for centers that are involved in the transfusion chain. The IRL role through Iranian national rare donor program is also to improve services and update the information on rare donors that have been identified at other centers. IRL provides services to 31 IBTO provincial blood donor centers, hospital blood bank laboratories and other medical centers, thalassemia centers, bone marrow & other transplantation centers. Annually, about 7000 to 10,000 samples from mentioned centers are referred to IRL. Department of Frozen blood processing and storage facility are part of the IRL.

When a patient with difficult blood type is identified in a hospital blood bank, the special serology laboratory is informed at the provincial blood centers. The physician's written request and 10 ml EDTA blood samples are forwarded to the laboratory. Preliminary work-up is performed at the provincial special serology laboratory. The central IRL in Tehran is contacted for consultation if necessary. If the serological test results are completed, the search to find matched RBC units is initiated at the provincial level. If matched RBC units cannot be found or if the serological identification needs more extensive work-up, the samples are sent to central IRL in Tehran for complete antibody identifications. The rare blood database is searched and if there is any frozen blood available, this blood after processing, thawing and deglycerolization is transported to the hospital via air or land transport supervised by a trained technologist. Matching donors also from data registry are contacted for donation if they are eligible to donate.

A summary of the activities of the central Iranian reference immunohaematology laboratory and frozen blood department pertaining to rare donor program is as follows:

• Retention rare donor and effective continuous relation (education, public relation, recalling & updating personal information) 

• Examining and resolving discrepancies in blood grouping complex, identifying alloantibodies and autoantibodies.

• Determining the principal blood group and other blood groups with different ways (Manual method, Automation & Molecular typing)

• Maintaining information on national bank of rare blood, keeping in touch with network of database of rare blood at the International Society of Blood Transfusion

• Participation in the evaluation and quality control of blood typing antisera according to international standards of WHO (NIBSC).

• Participation in External Quality Assessment Scheme (EQAS) with unknown samples from international centers

• Monthly production of RBC kits for determining antibody-screening strategy and detecting unexpected antibodies using frozen rare RBC aliquots.

• Training students and staff in specific serology.

**Facts and figures**

A total of 1300 rare donors were registered in this study. Fifty six individuals with rare Bombay blood group were identified in this survey. There were about 175 rare units in frozen state for transfusion. The most difficult types to find were Kpb negative, Rh null, K null, Jk(a-b-), S-s-U- . The average number of rare donor units used per year was close to 50 unites. Approximately 20 units of rare blood are delivered to the hospital via air or land transport annually. There are procedures in place to collect information when rare blood is forwarded and used. Special delivering form has been designed for this purpose.

**Challenges and important step to ensure successful establishment of a National rare donor program**

**Packaging and reliable transportation**

In order to transport the rare blood in liquid phase, a monitored temperature at 1-10 C should be maintained and standard commercially insulated and leak-proof boxes should be used. Several days may be required to keep a frozen supply of units of rare blood. As transport of such units over long distances presents a number of difficulties, special containers should be designed^1^. It is easier to ship frozen cells by glycerol and these can be stored in dry ice but deglycerolizing equipment should be available at the receiving facility. First of all, containers should be validated for their ability to maintain low-temperature storage for the required time period. Also, it is necessary to use data logger to record temperature information during transportation. 

Labeling of boxes should always be adequate. The labels should also have the full shipper address, full details of properly classified biological or infectious material, and the information on the container should indicate the contact party in case of delay in blood transport. For long-distance transport, the contract with specific airlines should be signed in order to transport the blood in a short period of time. The name and phone number of receiving center should be displayed on the outside of the container. 

Conformation of serological antibody testing and rare blood group identification are done at central IRL. It performs a crossmatch of any rare RBC units before shipping them in order to prevent misidentification or any discrepancies between IRL and medical center testing results. This might cause unnecessary delay or result in the loss of units. Therefore, the collection of a properly labeled pre-transfusion blood specimen from the intended recipient is critical for safe blood transfusion. The majority of hemolytic transfusion reactions arise from misidentification of patients or pre-transfusion specimen labeling errors^6^. 

**Standard Operating Procedures and use of a comprehensive Information Technology**

All centers have specific standard operating procedures designed to support the activities related to the handling of Immunohematology procedures and rare blood units. Because of sudden and unexpected requests for rare blood types, all centers are equipped with computers. Some of the data items which need to be collected and placed in a computer database are as follows:

- Donor personal information

- Rare donor database of donors and their antigen type
- Database for frozen unit inventory

Requests must contain sufficient information for accurate recipient identification. Each center is required to develop and apply policies and procedures for accurate patient identification and specimen collection. Problems might exist in the preparation, storage and transportation of rare blood. Survey shows few known failures in the systems have resulted in: wasting rare blood, receiving the rare blood in an unsatisfactory condition, or a delay in the delivery of rare blood.

Receiving centers are validated through acceptable performance of the procedure as well as proficiency of staff members.

**Engineering and technical maintenance**

Engineering staff are trained to follow proper storage requirements upon receipt of the blood from the remote blood centers during holidays and non-working hours. Temperature requirements during blood transport, monitoring of storage temperatures in refrigerators and freezers of blood component storage are available with continuous temperature monitoring devices that would be able to detect a temperature deviation before blood components might be affected. Daily checks of the temperature should be recorded by engineering staff in order to ensure proper operation of equipment and recorder.

Deviations from acceptable temperature ranges are annotated, dated and initialed on the temperature recording chart by the engineering staff who note the deviation, and appropriate correcting action is taken either in emergency or routine situations. The equipment should be so constructed as to sound an audible alarm when an abnormal situation is sensed.

**Facility and freezing condition**

Glycerol is most commonly used in RBC cryoprotectant and RBC units, in frozen state, are stored at 80°C (40% wt/vol glycerol, high-glycerol method). Frozen RBC expires after 10 years, but AABB standards permit storage of frozen units for 10 years ^7^.

Frozen units must be thawed, and prior to transfusion, the glycerol must be removed. The use of thawed RBCs is limited to 24-hour expiration when stored at 1°C to 6°C because of concerns related to potential bacterial contamination when a functionally open system is used to add and remove the glycerol. Thus, the critical care specialist should understand the transfusion planning purposes and notice that preservation of post-thaw rare blood unit is limited to hours, whereas frozen rare blood units may be preserved and stored for many years. The recently developed closed systems have an extended expiration date to 14 days for thawed, non– leukocyte-reduced, and deglycerolized RBCs when stored at 1°C to 6°C ^8^. For extended storage of units in the case of antigen negative rare donor that is used in autologous or allogeneic transfusion, cellular components of RBCs are frozen, and for this reason, it is considered to provide appropriate equipment and various devices such as refrigerator and freezer. However, loss of absolute red cell mass which is resulted from red cell damage, will occur. 

High-glycerol freezing method is utilized, the 57.1% wt/vol high-glycerol, slow-freeze technique.

During, thawing and deglycerolizing RBCs the freezing canister in which the RBC unit has been stored is placed in a 37 °C dry heaters, or, after overwrapping, is placed in a 37 °C water bath. When the red cells have been frozen in the primary blood container, then the container is thawed at 42 °C. Thawing is completed within 40 minutes ^9^^,^^10^. 

Different situation requires different numbers of staff and different skill levels. Type of patient surgery, either routine or emergency, and number of rare RBC units will determine the type of staff and resources. Moreover, performing procedures require knowledge and skills. 

**Employee awareness and communication between regional blood centers**

One of the key components of quality management and quality improvement is the employee knowledge and awareness, then a key component of employee performance is training. To do the job well, staff must be properly trained to fulfill responsibilities. Clear communication is an important issue in different blood centers that are involved in requesting blood for a patient with rare blood types or receiving a rare blood shipment. An assigned person in each blood center is responsible for communicating the relevant information, including physician request, the patient’s blood sample, packaging for shipping and receiving samples.

**Technologist Education and Training in Advanced Immunohematology**

Different donor centers may require different numbers of staff and skills in rare blood collection, processing, compatibility testing, storage, or distribution of rare blood. All personnel are required to have educational background, job training and experience to perform their duties.

The type of staff resources will be determined by the donor center’s immunohematology activity level, number of difficult samples received and number of medical centers in the region which need rare blood services. Specific licensing requirements need to be provided by technologists who are practicing as professionals in Immunohematology reference serologic testing need to be provided. Job descriptions is enhanced and reflect employment requirements by job title. 

At management level, staff members supervise the daily operations of the unit, write procedures; train the serology staff, and enforce compliance with applicable procedures, standards and equipment. Education and training of the employees are applied in the preparation of rare blood and performance of Immunohematology standards.

Employees with a college degree in one of the sciences such as laboratory science can be trained successfully to perform specific serologic tests. Staff should be promoted and provided opportunities to attend continuing education seminars on topics in serologic and related fields. In numerous states, continuing education is required for the renewal of licenses.

**Financial and Administrative Support by Higher Management**

Due to the cost of equipment and consumables used in rare blood banks, especially freezing and storage devices, budgeting in rare blood services need special attention and dedication by higher management and top managers of the blood- service systems. So, the government is required to provide financial support to blood banks. According to the study of Geralyn et al., the cost of collecting, testing, storing and transporting blood to hospital transfusion service in the United States is approximately $925 to $1150 per RBC unit^11^. According to The American Rare Donor Program (ARDP), the cost of special antigen typing will rise to $500 - $1200 per each unit. Meanwhile, nearly $1940 to $2800 may be added to units imported to the United States ^12^. Thus, the cost of a rare unit of blood in the United States ranges from approximately $1148 to $1373. 

Cost of preparing and handling a rare blood unit may differ between countries according to the policies of blood centers. To date, the rare blood services for patients are provided free of charge in Iran and all is paid by the government, including transportation charges.

## Discussion

 During the past decades, interest in national rare donor program has increased in a significant manner worldwide. This is especially true in developed countries. In developing countries, however, the experience is not a lot. We report the results and challenges of the rare donor program, which was initiated in 2009. 

All activities used at IRL was based on those reported previously by different international organizations. In the current year, database consisted of 1300 personal information of rare donors and 308 RBC units in frozen state. As the proportion is similar to those reported by different countries, it is important to notice that this is the initial phase of our program, and we have evidence that this number will increase in the following years, as our collection program of donor and rare units improves.

According to the study of Woodfield, many areas of the world have problems in the storage, transportation and financing of rare blood^1^. Between January 2005 and June 2006, there were approximately 51 000 active donors in the rare donor registry. Rare units were not always available to fill a transfusion request. Flickinger examined the effect of ARDP in filling requests for high incidence antigen-negative RBC units for patients with sickle cell disease. She reported that 88% of 141 requests were completely or partially filled. The top 3 requested phenotypes for this patient population were U−D+, Js(b−), and U−D− ^13^. The study of Goodell et al. focused on patients with routinely encountered red cell antibodies. Seltsam et al. studied blood banks in Germany, Switzerland, and Austria to identify transfusion support^14^^, 15^.

During the 20-month survey period, 52 patients with antibodies to high-frequency antigens were hospitalized on 56 occasions (49 routines, 3 urgent, and 4 unknown). The top 5 antibody specificities identified included anti-Kp^b^, anti-Vel, anti-Lu^b^, anti-Yt^a^, and anti- Co^a^. Insufficient blood was offered to 40% (23/56) of transfusion cases. Procedures continued without transfusion in 10 cases. Antigen-positive RBCs were transfused in 8 cases and led to 5 cases of delayed hemolytic transfusion. The authors note that the additional time is required to achieve these rare units and recommend that additional group O donors be typed for blood for Kp^b^, Vel, Lu^b^, and Yt^a^. Thus, for a patient with previously identified multiple alloantibodies, the critical care specialist may consider transfusing alternative units such as D-positive units to a D-negative patient or antigen-positive units for those antibodies that are no longer serologically detectable until compatible units are provided by the ARDP. One can then evaluate the patient for the presence of intravascular and extra vascular hemolysis^16^. Nonetheless, one should not withhold transfusion from a patient whose life depends on urgent transfusion.

If an autoantibody appears to be present and one is uncertain whether underlying alloantibodies are present, transfusing blood matched for ABO, Rh (D, C, and E), and K (Kell) should be considered. Additional antigens may be matched if time permits. Selecting “least incompatible” blood for transfusion is unreliable ^17^.

## CONCLUSION

 According to the results and reports of different countries in ISBT working party 2012 for rare donors, the majority of member countries have nationalized rare donor program. The number of donors in database and number of frozen rare cells are different in various countries. The definition of a rare blood 1/1000 is the same in all member states, except 1/250 in South Africa. Also, the most difficult rare blood types differ in *various regions*: in China (Rhnull; D - - ), Finland (Vel neg; Oh; hr S −), France (U-; Fy(a−b−); Vel− ; Rhnull; D - -; Hr−; HrB –) and in Iran (D - -; E− c− K- Jk(b−); E−c− K− Jk(b−) Fy(b-); C− E− Jk(b−) S− M−; E− C− c− e− ). Rhnull has been reported in 9 countries: Spain, Switzerland, Taiwan, Netherlands, Israel, India, France, Germany and China. D --, U- and Ko have been reported in 6 countries. Moreover, the availability of especially effective reagent is controversial in many countries^11^.

Finally, in order to improve rare donor program, we have to overcome economic and other issues. So, the government should allocate sufficient budget for initial establishment and continuous improvement program of rare donor services of Iranian National Rare Donor Program. It is necessary to put a heavy emphasis on continuous education, training and improving knowledge of technologists with special reference to serology in various provincial blood donor centers. The importance level of services offered to patients with difficult samples by rare donor program should be promoted to prevent transfusion-related adverse effect. To overcome the lack of experience in using special immunohematology procedures as well as technical and human errors in reporting test results, effective contact and collaboration with international immunohematology reference laboratory for technical help and testing confirmation is required. Easy access to expensive internationally accepted anti-sera availability of automated machines, supplies and consumables for glycerolization and deglycerolization of rare RBCs should be provided. Moreover, there is a need for access to sufficient storage area and good environmental condition, sufficient number of -80°C standard freezers for longer blood storage, empty back up freezer, controlled access area temperature, controlled environment (-18 °C -22°C) by central heating and cooling system, central 24-hour temperature control, and alert system for any electrical failure. Finally, serious difficulties that occur in delivery of blood by Air and Land (bureaucratic procedures /ticket purchase / cancellation of flights/ flight delays), due to lack of experienced private companies, should also be considered.
